# The Sanitarian

**Published:** 1881-12

**Authors:** 


*The Sanitarian.*—A monthly magazine devoted to the preservation of the health, mental and physical culture. Edited by A. N. Bell, M. D., and T. P. Cerbally, M. D., 8 Spruce Street, New York. $3.00 a year in advance.

This useful and instructive monthly is conducted in a manner which maintains the reputation it has for years merited, and is well worth a place in medical literature.

